# Adenomatoid Tumor of the Tunica Albuginea in a Boy: A Case Report and Literature Review

**DOI:** 10.1155/2015/935193

**Published:** 2015-05-27

**Authors:** Kaimin Guo, Runhui Tian, Lingyun Liu, Congqi Du, Fubiao Li, Hongliang Wang

**Affiliations:** ^1^Department of Andrology, First Hospital of Jilin University, Changchun, Jilin 130021, China; ^2^Department of Psychology, First Hospital of Jilin University, Changchun, Jilin 130021, China

## Abstract

Adenomatoid tumors (AT) are the most common paratesticular neoplasms and account for approximately 30% of all paratesticular masses. Most of them occur in the third or fourth decade and present as well-defined firm and painless masses. We report here a case of adenomatoid tumor from tunica albuginea. This patient is a 12-year-old boy with left testicular pain for 6 months. Scrotal ultrasonography revealed a solid mass of paratesticular origin. The histology and immunohistochemistry confirmed the final diagnosis. A right tumor resection was performed. Because of its rarity, the clinical and histopathologic appearance is seldom illustrated. Here we present a case report and a comprehensive literature review with the objective of providing useful information on this entity.

## 1. Introduction

Adenomatoid tumors (AT) are benign tumors most commonly occurring in the male and female genital tracts. Extragenital AT are rare and have been found in the adrenal glands, heart, mesentery, lymph nodes, and pleura [1]. When the mass arises from the tunica vaginalis or tunica albuginea, sonographic findings may distinguish it from a peripheral testicular tumor [2]. The structural growth pattern is atypical of benign neoplasms, as they are generally not encapsulated, and tumor elements are commonly present between the structures of adjacent tissues and may show clear-cut infiltration [3]. Considering most intratesticular tumors are malignant, we report a rare case and management of an AT originating from tunica albuginea.

## 2. Case Report

A 12-year-old boy presented to the andrology department in June 2013 with left testicular pain that he had had for 6 months. There was no history of recent trauma, infection, hydrocele, or undescended testis. He denied having any urinary or constitutional symptoms. Physical examination revealed a hard, tender, 10 mm × 10 mm × 8 mm testicular nodule in the superior aspect of the left testis. The contralateral testicle was normal.

**Figure 1:**
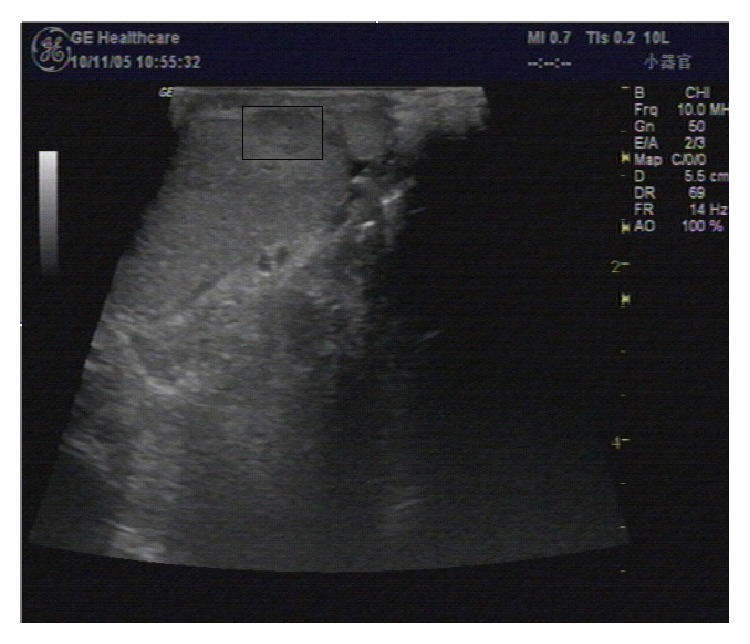
Scrotal ultrasonography of AT: scrotal ultrasound scan revealing a 10 × 8 mm, hyperechoic solid mass in the upper pole of the left testicle.

Scrotal ultrasonography (8 to 12 linear array transducer, LOGIQ P5, GE Healthcare, New York, New York State, USA) revealed 8 mm × 10 mm, hypoechoic homogeneous solid mass with unclear margin at the junction of the epididymis and left testis (Figure 1). Serum tumor markers, including alpha-fetoprotein, beta-human chorionic gonadotropin, and lactate dehydrogenase, were all within normal limits. All preoperative laboratory tests, including complete blood count, biochemistry, and chest X-ray, were normal. A pelvic computerized tomography (CT) scan was negative for retroperitoneal metastasis. The provisional diagnosis was paratesticular tumor, with the possibility of benign nature.

The patient was then referred to our institution for surgical treatment. The left inguinal approach was established and the left spermatic cord was identified. On visual inspection, the tumor arose from the tunica albuginea protruding into the testis parenchyma. Intraoperative frozen-section biopsy showed benign tumor from tunica albuginea. Subsequently, a right tumor resection including removing a portion of tunica albuginea was performed.

**Figure 2:**
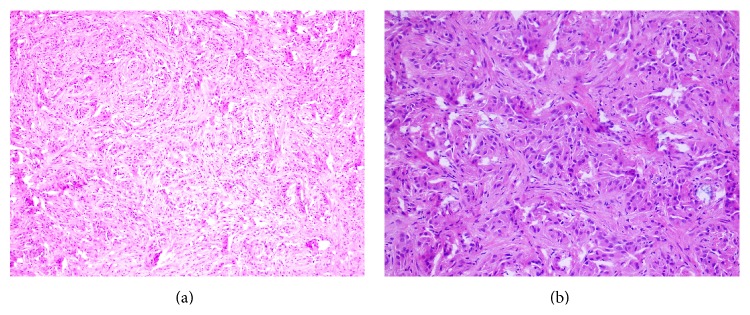
HE staining of AT: (a) hematoxylin-eosin stain of tumor biopsy showing tumor cells lined in irregular, glandular pattern, and fibrous tissue proliferation in stroma with unclear margins (×100); (b) the neoplastic cells had round to polygonal outlines, moderate to abundant pale to vacuolated cytoplasm with round or oval nuclei. No mitoses were seen (hematoxylin-eosin, ×200).

**Figure 3:**
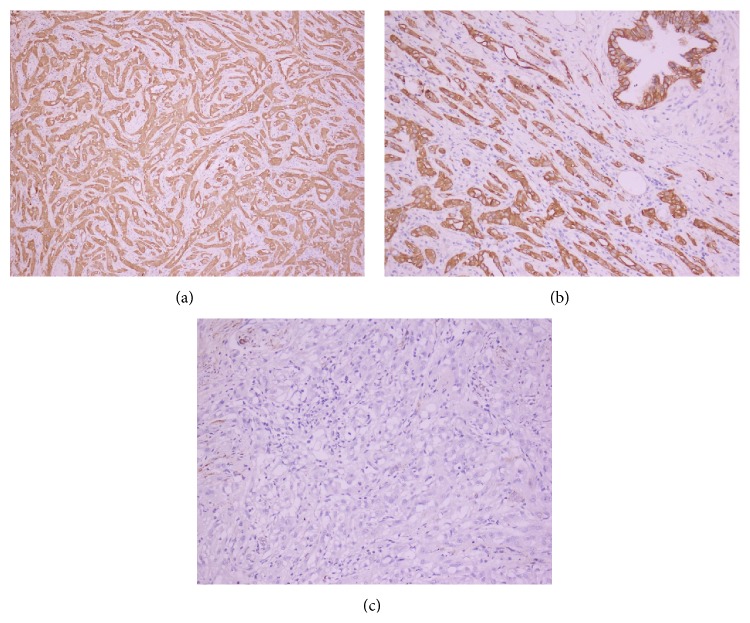
Immunostaining of adenomatoid tumors. (a) Calretinin (+); (b) cytokeratin (+); (c) vimentin (+) (×200).

Final histological examination confirmed the diagnosis of AT from the tunica albuginea (Figure 2). Immunohistochemical analysis revealed the tumor cells were positive for calretinin, cytokeratin, and vimentin (Figure 3). The postoperative course was uneventful. After 12 months of follow-up, the patient was asymptomatic without any evidence of local recurrence.

## 3. Discussion

AT are the most common paratesticular neoplasms and account for approximately 30% of all paratesticular masses [4]. It was firstly described by Golden and Ash in 1945 [5]. The epididymis is the most common site of involvement. The origin from the testicular tunica is estimated 14% of AT [6]. We searched relevant case reports published in English that were available in full-text and found only 7 related cases (Table 1).

In our review, AT from tunica albuginea usually occur in the third or fourth decade and present as well-defined firm, painless masses that have been noted for several months to 1 year or discovered incidentally on physical examination. Most of them are asymptomatic and only two patients complain of dull pain of testis or scrotal discomfort [7]. Right-sided tunica albuginea is easily involved (right/left: 6/2), and lower pole of testis is commonly located. They are usually small in size rarely exceeding 5 cmm (range is 0.5–5.0 cm). The origin of this tumor is unknown, but some are considered to be a reaction to either injury or inflammation, and others are thought to be of mesothelial origin [8]. 7 cases of AT from tunica albuginea had no history of scrotal trauma or testis infection except one case reported pulmonary tuberculosis. We conclude the occurrence and progression of AT from tunica albuginea have no relationship with past injury and inflammation.

Ultrasound of the scrotum reveals a well-defined interface between the tunical mass and the displaced testicular parenchyma [9]. Kolgesiz et al. described a maneuver that radiologists examine the mass and the testis on a sagittal view with the ultrasonic probe in one hand, while pushing the testis downward with the other hand which could facilitate differentiating the mass as being extratesticular in location [10]. Magnetic resonance imaging (MRI) allows characterization of scrotal masses as being intratesticular or extratesticular and can demonstrate various types of lesions and tissue, including cysts or fluid, solid masses, fat, and fibrosis [11]. The depiction of the thin low-signal well-delineated zone between the mass and the adjacent testicular parenchyma, corresponding to the tunica albuginea, helped to indicate the origin of the mass [12]. In our case, a hypoechoic, homogeneous, solid mass with unclear margin adjacent to the tunica albuginea was noted by ultrasonography. So the preoperative diagnosis of testicular tumor was established.

The imprint cytology and fine needle aspiration cytology (FNAC) have been applied to confirm the diagnosis [12]. Microscopically they have two major elements: epithelial-like cells and a fibrous stroma. Epithelial cell arrangement allows for recognizing three patterns: plexiform, glandular, and angiomatoid. Stroma may consist of loose or dense collagen tissue with hyalinization and is found in variable amounts. The cytological origin of the AT is controversial and the cells are cytokeratin 5/6, calretinin, and vimentin positive, as we could show in the present case report, and epithelial markers of factors VIII and CD34 negative [13], indicating the mesothelial nature of the lesion. Thus, cytokeratin 5/6 and calretinin are useful to distinguish between mesothelial tumor and seminoma.

Orchiectomy has traditionally been the gold standard for the treatment of testicular tumors in children. Tumor resection appears to be the treatment of choice only when a benign lesion confirmed. But in our review, we found only one case underwent partial orchiectomy and one case performed tumor resection. The rest of cases underwent orchiectomy. Both Garg and Monappa established the diagnosis of seminoma preoperatively by FNAC [7, 14], while Barry, Lee, and Evans described cases with ill-defined lesions and unclear borders between masses and the testis, which made the malignancy not be excluded [9, 12, 15]. In our present case, even if the tumor demonstrated unclear border with testis in ultrasonography and protruded into the testis parenchyma in operation, the testis and the epididymis were not involved. After consulting with an experienced pathologist intraoperatively and preserving the patient's fertility potential, removing the tumor with clear margin and suturing the tunica albuginea were considered. AT have not been known to ever recur or show malignant degeneration. According to literature, no cases of malignization, metastasis, or relapse after removal have been reported.

## 4. Conclusions

This case highlights the difficulty of accurately diagnosing a paratesticular mass preoperatively when the origin of the mass cannot be established before excision. We report this case for its rarity of origin and occurrence in a young boy. So this benign tumor should be borne in mind and testis should be spared as possible as we can.

## Figures and Tables

**Table 1:** Characteristics and clinical course of published cases of AT from tunica albuginea.

Case number	Age	Symptom and duration	Location	Size	Treatment	Past history
1 [7]	40	Dull pain of right scrotum for 1 year	The lower pole of the RT	4 × 3.5 cm	RO	Previous seminoma by FNA

2 [9]	40	A painless, rapidly growing mass for 1 year	The lower pole of the RT	5 cm	RO	Negative

3 [10]	45	A painless palpable mass for 1 month	Anterior surface of RT	0.5 × 0.7 cm	Tumor resection	Negative

4 [11]	27	A painless palpable mass of left scrotum	The lower pole of the LT	1.0 cm	Partial orchiectomy	Negative

5 [12]	36	A painless palpable mass for 2 months	The lower pole of the RT	0.8 × 0.7 cm	RO	Pulmonary tuberculosis

6 [14]	40	A painless palpable mass for 1 year	The lower pole of the RT	4 × 3 cm	RO	Negative

7 [15]	44	A painless palpable mass for 1 year, enlarging for 3 months	Midposterior aspect of the RT	0.7 × 0.6 × 0.5 cm	RO	Negative

Our case	12	Left testicular pain	Upper pole of the LT	0.8 × 1.0 cm	Tumor resection	Negative

RT: right testis; LT: left testis; FNA: fine needle aspiration. RO: radical orchiectomy.

## Abbreviations

AT: Adenomatoid tumors
FNAC: Fine needle aspiration cytology
RT: Right testis
LT: Left testis
RO: Radical orchiectomy.
